# Supplementary figures and images for: The binding modes of brazilin and hematein from Caesalpinia sappan L. to Cutibacterium acnes lipase: Simulation studies
Source: PLoS One. 2025 Mar 4;20(3):e0318706. doi: 10.1371/journal.pone.0318706 (PMC11957764; doi:10.1371/journal.pone.0318706)

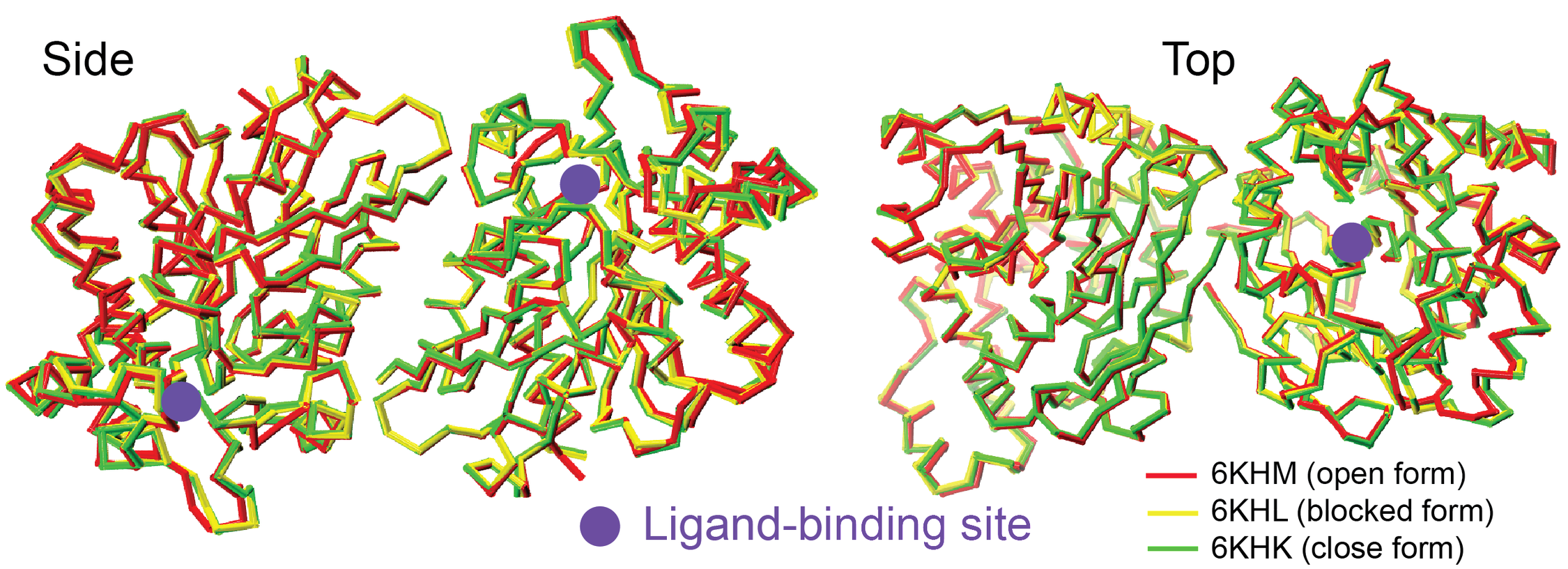

Supplement: S1 Fig — (TIF) [file pone.0318706.s001.tif]

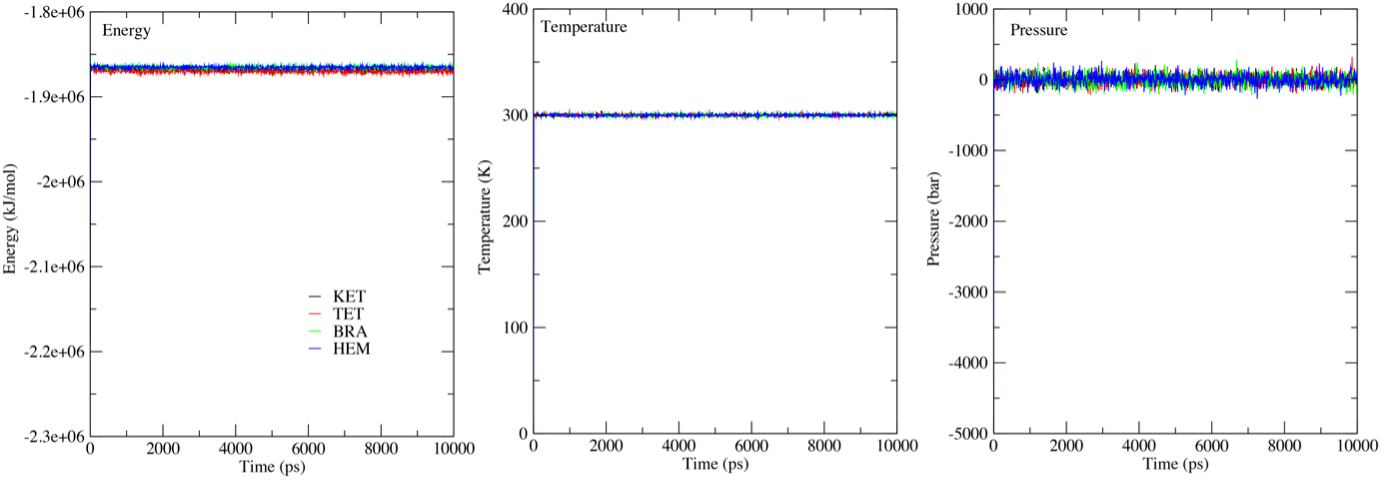

Supplement: S2 Fig — (TIF) [file pone.0318706.s002.tif]

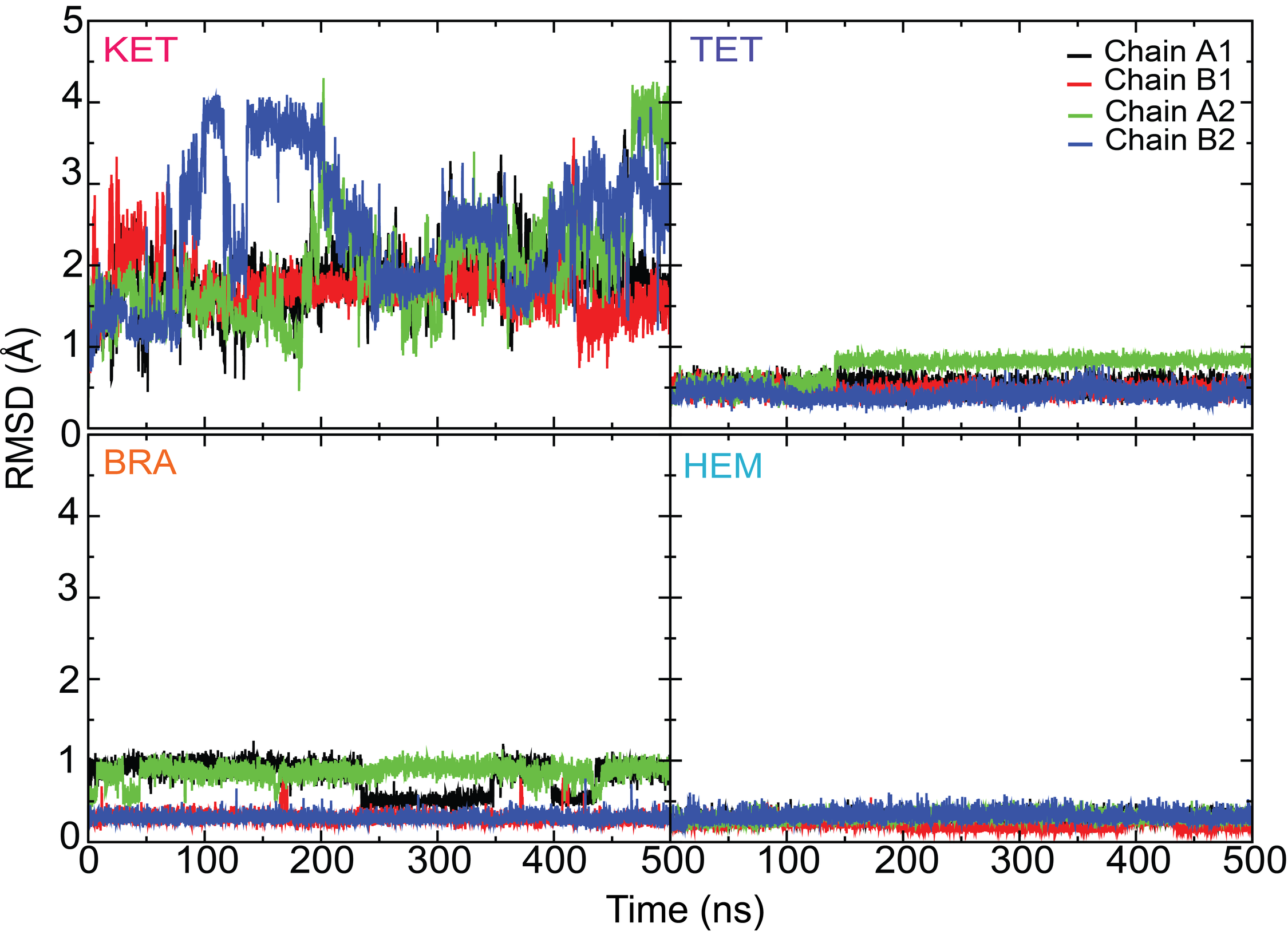

Supplement: S3 Fig — (TIF) [file pone.0318706.s003.tif]

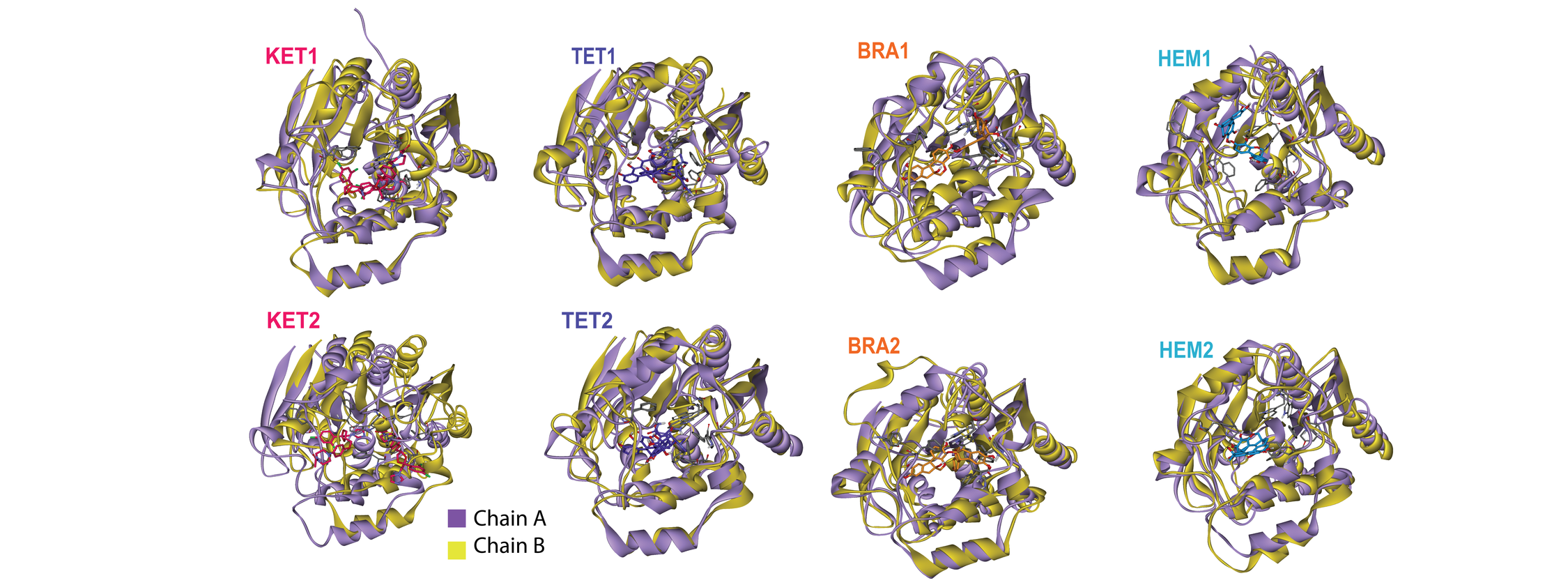

Supplement: S4 Fig — (TIF) [file pone.0318706.s004.tif]

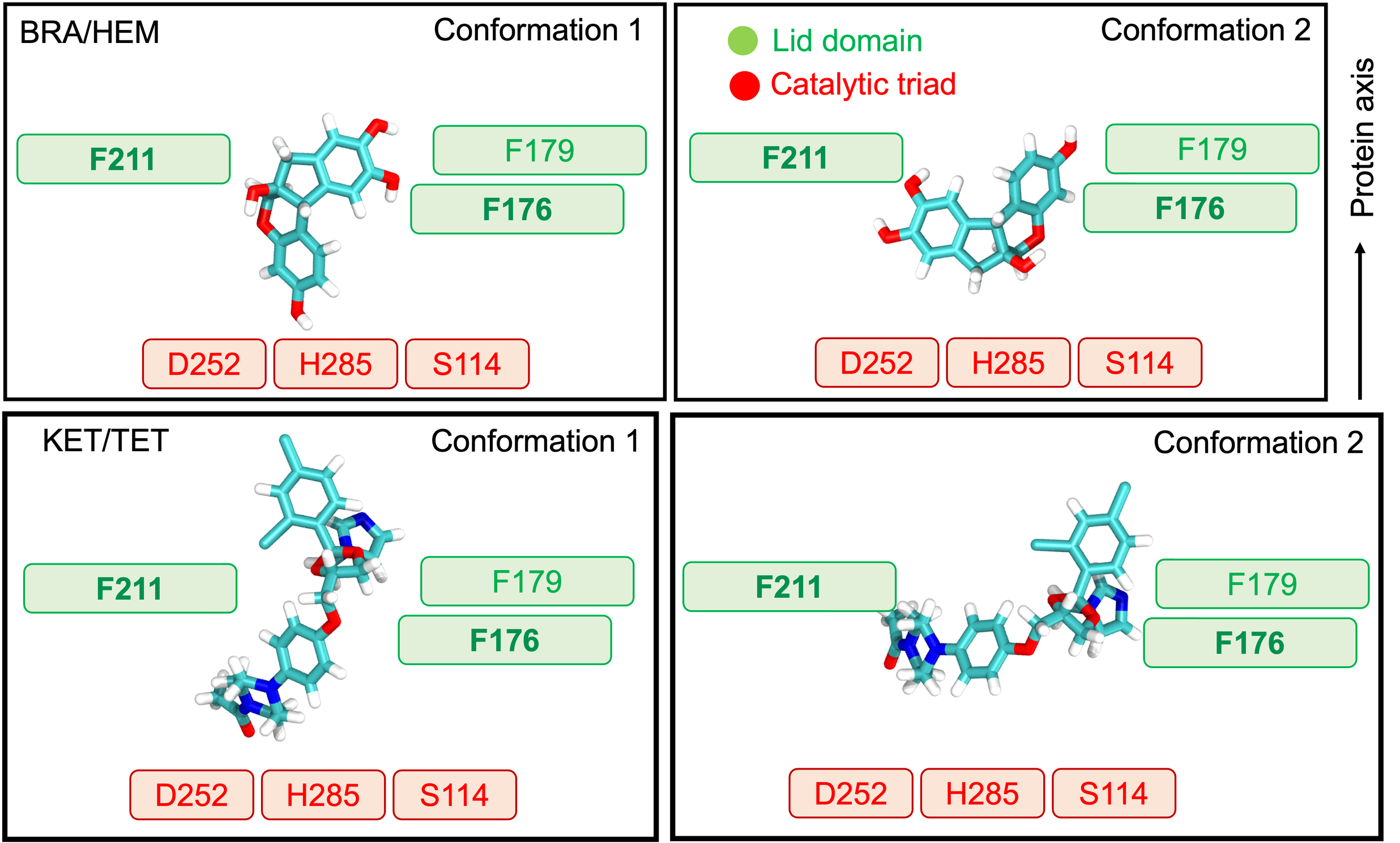

Supplement: S5 Fig — (TIF) [file pone.0318706.s005.tif]
